# An Unusual Presentation of Villoglandular Papillary Adenocarcinoma in a Perimenopausal Woman

**DOI:** 10.7759/cureus.54374

**Published:** 2024-02-17

**Authors:** Priya R Nair, Saunitra A Inamdar, Minal A Kalambe, Aakanksha Dewangan

**Affiliations:** 1 Obstetrics and Gynaecology, Datta Meghe Medical College, Datta Meghe Institute of Higher Education and Research, Nagpur, IND

**Keywords:** endocervicitis, malignancy, radical hysterectomy, perimenopausal woman, cervical cancer, villoglandular papillary adenocarcinoma

## Abstract

Villoglandular papillary adenocarcinoma (VPA) or villoglandular adenocarcinoma (VGA) is a rare but well-recognized subtype of cervical carcinoma. It exhibits a favorable prognosis, particularly within the childbearing age group, and is considered a rare manifestation of mucinous adenocarcinoma typically observed in individuals of reproductive age. In comparison to other adenocarcinomas, VPA generally demonstrates a more optimistic prognosis. This report details the case of a 46-year-old perimenopausal woman who presented with complaints of irregular menses and a protruding mass from the vagina. Upon examination, an exophytic growth was identified, replacing the cervix. A biopsy confirmed the diagnosis of VPA. Subsequently, the patient underwent a radical hysterectomy, followed by post-operative radiation therapy.

## Introduction

Villoglandular adenocarcinoma (VGA) of the cervix is a subtype discovered by Young and Scully in 1989 [1]. In 1994, it was officially recognized by the World Health Organization (WHO) in the histological typing of uterine cervical cancer [2]. Mucinous adenocarcinoma accounts for 4-9% of endocervical adenocarcinomas, of which VGA is a very rare subtype [3]. While carcinoma of the cervix is typically associated with the elderly, VGA is primarily observed in females of reproductive age, with a mean age of 33 years [3]. The typical histopathological features of this adenocarcinoma include villoglandular and papillary structures with a fibrovascular core of inflammatory cells lined by columnar cells with hyperchromatic nuclei [4]. Generally, VGA exhibits a more favorable prognosis compared to other adenocarcinomas, which facilitates the preservation of fertility in younger populations through conservative management [1,5]. This case report details the occurrence of VGA in a perimenopausal woman, a rare finding. Given her age and parity, a decision for radical surgery was undertaken.

## Case presentation

A 46-year-old female, para 2, live 2, had chief complaints of white vaginal discharge and felt something was coming out of the vagina in the last six months. She presented to the outpatient department accompanied by her relatives on June 10, 2021. She was perimenopausal with irregular menses every two to three months, lasting three to four days with average flow. The last menstrual period (LMP) was on March 30, 2021.

She attained menarche at 12 years of age and married at 14 years of age, with a monogamous relationship. She had no use of any type of contraception during her reproductive period. Both children were home-delivered with the help of dai, with the last childbirth 23 years back. Later on, the patient was tubectomised also 23 years back. There was no major medical or surgical history except the tubectomy operation. The patient had never smoked or taken drugs or alcohol, and her family history was not suggestive of inherited diseases or carcinomas.

On general examination, pulse was 86/min, heart rate was regular, and blood pressure was 120/70 mm Hg. There were no pallor, icterus, and edema feet. The chest was clear, and heart sounds were normal. As per an abdomen examination, soft, non-tender, and no hepatosplenomegaly were noted. As per speculum examination, there was evidence of exophytic cauliflower-like growth, which was fragile, papillary, bleeding on touch, and with copious mucoid discharge. It was approximately 7 x 5 cm in size, involving the whole of the cervix. The vagina was pale, and rugosity was decreased, with no vaginal involvement, with the upper and lower vagina free. As per a vaginal examination, the same mass was felt vaginally. The isthmic portion and the upper vaginal mucosa were free, and the uterus was normal-sized. As per a rectal examination, rectal mucosa was free and parametrium was free on both sides.

**Figure 1:**
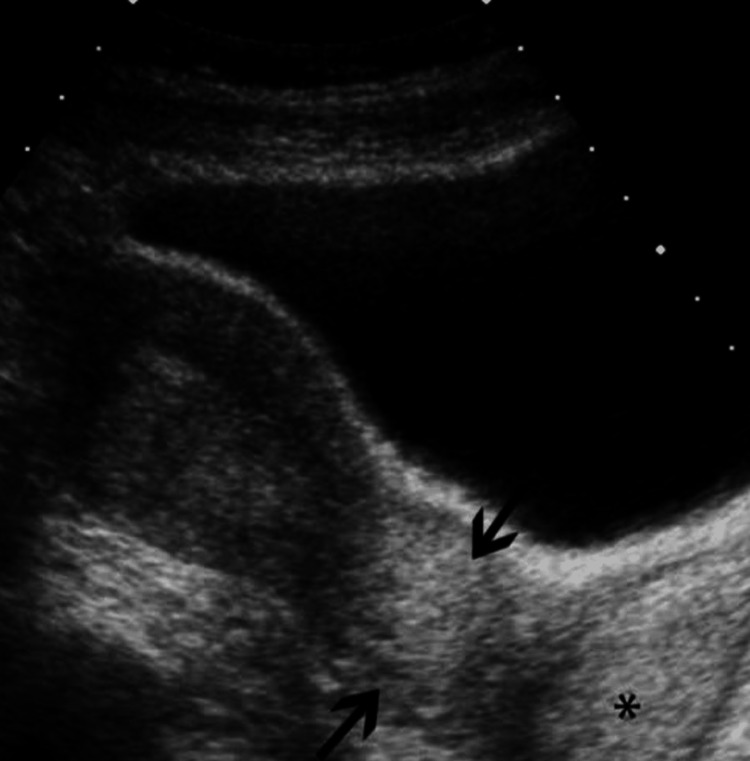
Ultrasonography showing bulky uterus with thickened lower uterine segment and cervix The arrows show a thickened lower segment of the uterus, and the asterisk shows a thickened cervix.

Having in mind these clinical findings, a diagnosis of cancer cervix stage IB3 was initially made. A differential diagnosis of herpes infection of the cervix (papilloma) was also made. A Papanicolaou (Pap) smear was taken, and the patient was posted for examination under anesthesia and for a cervical biopsy. Ultrasonography was suggestive of a thickened lower uterine segment and cervix with a bulky uterus (Figure 1).

**Figure 2:**
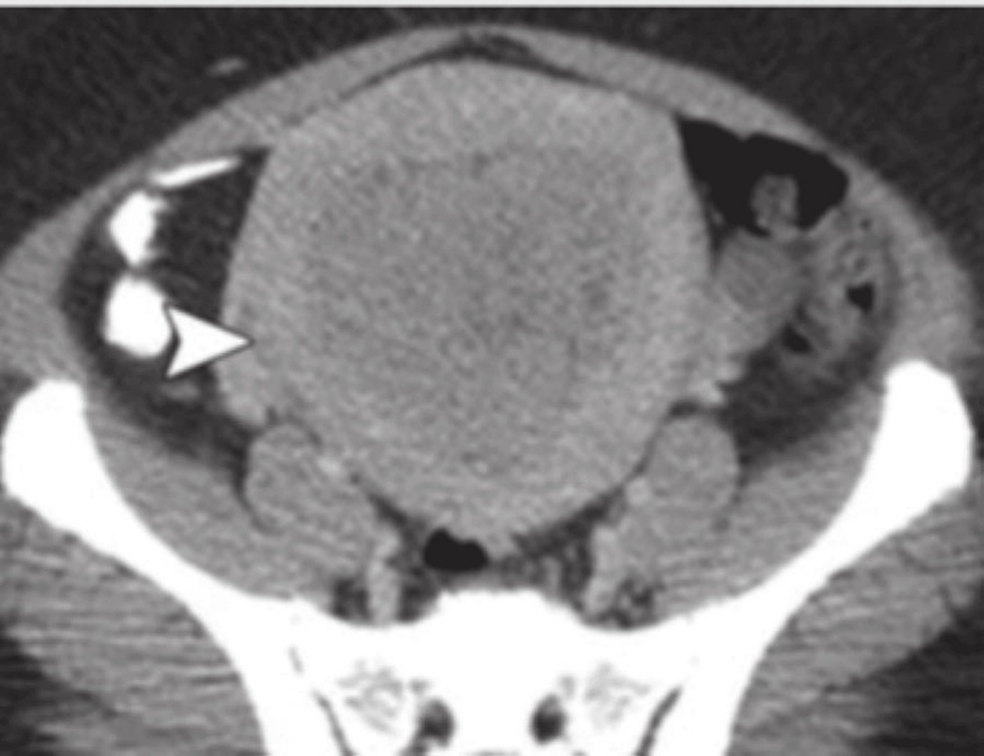
Axial CT of the patient The arrow shows the bulky uterus with no parametrial invasion. CT: Computerized tomography

**Figure 3:**
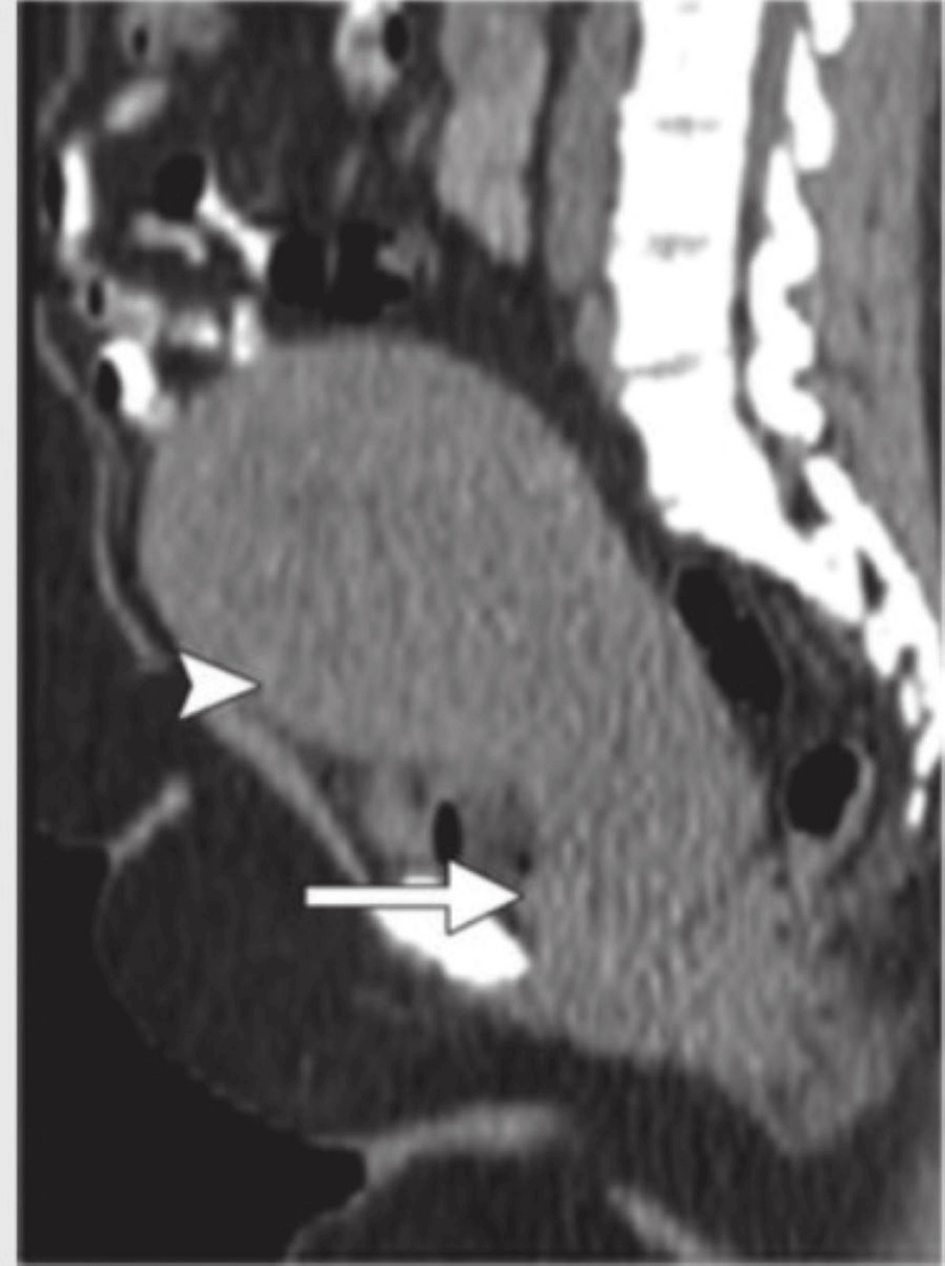
Sagittal view of carcinoma cervix showing bulky uterus with mass in cervix The top arrow shows the involvement of the lower uterine segment and the bottom arrow shows the cervix.

Contrast-enhanced computerized tomography (CECT) of the abdomen pelvis was suggestive of a heterogenous mild to moderately enlarging polypoidal mass arising from the anterior wall of the cervix involving the lower uterine segment, suggestive of malignant neoplastic etiology (carcinoma cervix stage IIA) as shown in Figure 2 and Figure 3.

PAP report was suggestive of atypical squamous cells of undetermined significance (ASCUS). The histopathology report of cervical biopsy was suggestive of chronic papillary endocervicitis with cervical glandular hyperplasia.

Considering the age and disparity in the CECT report and clinical examination, the patient underwent Wertheim’s radical hysterectomy with lymph node dissection. The investigation results were within normal limits with complete blood count (CBC) values as hemoglobin (hb) at 11 gm/dL, white blood cells (WBC) at 7,300/mm^3^, and platelets at 250,000/mm^3^.

The patient was pre-operatively optimized with bowel preparation for three days and a semisolid diet. Her radical hysterectomy was done in a tertiary care institute by a senior oncosurgeon, with the help of a senior gynecologist, under spinal anesthesia with an epidural top-up. She was given pre-operative antibiotics, analgesics, and antiemetics. An infraumbilical vertical incision was cut, and the abdomen was opened in layers. A stepwise radical hysterectomy was done. The uterus, cervix, and upper cuff of the vagina, along with parametrial tissue, were removed. External iliac and obturator lymph node dissection was done, and the specimen was sent for histopathology.

Intraoperative blood loss was approximately 700-800 mL. The patient was transfused with two packs of red blood cells (RBC) post-operatively. Antibiotics and analgesics were continued post-operatively for seven days. Low molecular heparin was given for thromboprophylaxis for three days post-operatively. The patient’s recovery post-operative was uneventful. The histopathology report of the specimen sent was suggestive of VPA of the cervix. Lymph nodes were free of infection or malignancy. The lower uterine and vaginal cuff were free of malignant cells.

The patient was discharged on day 10 of the post-operative period and advised to follow up after three weeks for radiotherapy sessions. She was advised to refrain from heavy weight lifting, infection, and sexual intercourse for two months. On follow-up, the patient was referred to the oncology department for radiotherapy. One year follow-up with regular investigations and ultrasonography showed normal reports. The patient's compliance was good.

## Discussion

The prevalence of adenocarcinoma is 10-20%, and it is the second most common carcinoma of the cervix following squamous cell carcinoma [1]. Jones et al. cited an opinion on the association of the use of oral contraceptive pills with villoglandular papillary adenocarcinoma (VPA), but a definitive association has not yet been found [3]. The etiology of VPA is not well established [4]. Treatment ranges from cone biopsy to simple or radical hysterectomies, including or excluding lymph node dissection, along with pre-operative or post-operative radiotherapy, depending upon the age and extent of the spread of the tumor [6-8]. The prognosis of this tumor can vary depending on various factors of patients, including age, extent of spread, and type of method used for management but most cases have very good prognosis [1,9,10]. While some cases have rapidly deteriorated, a good prognosis mostly accounts for the early detection and early staging of the tumor [10,11,12]. Non-involvement of lymphovascular invasion and nodal metastases is usual. In our patient, the early staging and considering the age of the patient, the decision of radical hysterectomy with pelvic node dissection followed by radiotherapy was taken. The post-operative period of the patient was uneventful, with rapid recovery in the patient's health.

In a case report by Salek et al., the patient was 29 years old, para 1, with a history of postcoital bleeding. Her histopathological examination of the cervix was suggestive of VPA, and magnetic resonance imaging (MRI) was suggestive of cervical mass with parametrial involvement and no pelvic or retroperitoneal lymph node involvement. The diagnosis was stage IIB carcinoma cervix. The patient underwent chemoradiotherapy as the surgical management level had crossed. She responded well to the management [13]. Another case report by Takai et al. mentioned the management of a pregnant primigravida at nine weeks gestation with vaginal bleeding with exophytic growth. A biopsy of the growth was taken, which revealed VPA with no lymphovascular space involvement. She underwent cone biopsy at 16 weeks gestation and continued pregnancy till term. The cone biopsy was not suggestive of cancer cells [14].

Management of VPA varies according to the age, parity, and the staging of carcinoma. Young and Scully recommended a histopathological examination of the specimen for villoglandular pattern [1]. There can be other adenocarcinoma, such as serous papillary adenocarcinoma, in which the cervix presents with finer, irregular, and cellular papillae than VPA. The adenocarcinoma subtype of the clear cell variant has more cellular atypia with high mitotic activity and pathognomic features of psammoma bodies. Adenosarcoma and adenoma malignum, which are rare variants, should also be considered in the differential diagnosis of VPA [15]. The prognosis is usually good due to the early staging of VPA and the absence of lymphovascular space involvement and lymph node metastasis.

The fertility-conserving procedure is suggested in most cases. However, in the present case, the patient was middle-aged with her family completed. Therefore, surgical management with hysterectomy was planned.

## Conclusions

VPA of the cervix, even though rare, can be diagnosed at an early stage based on the symptoms, clinical examination, and histopathological examination of the biopsy specimen. It is managed well with a very good prognosis for the patient. The staging, age, and parity of the patient should be considered before planning the management. Early stages can be treated conservatively with cone biopsy and follow-up, but advanced cases should have a multidisciplinary approach required for proper treatment and follow-up. Counseling to the patient and relatives regarding the prognosis of carcinoma is required to have better post-intervention outcomes.
